# 16S rRNA Gene Pyrosequencing Reveals Bacterial Dysbiosis in the Duodenum of Dogs with Idiopathic Inflammatory Bowel Disease

**DOI:** 10.1371/journal.pone.0039333

**Published:** 2012-06-15

**Authors:** Jan S. Suchodolski, Scot E. Dowd, Vicky Wilke, Jörg M. Steiner, Albert E. Jergens

**Affiliations:** 1 Gastrointestinal Laboratory, Department of Small Animal Clinical Sciences, College of Veterinary Medicine and Biomedical Sciences, Texas A&M University, College Station, Texas, United States of America; 2 Molecular Research, Shallowater, Texas, United States of America; 3 Veterinary Clinical Sciences, College of Veterinary Medicine, University of Minnesota, St. Paul, Minnesota, United States of America; 4 Veterinary Clinical Sciences, College of Veterinary Medicine, Iowa State University, Ames, Iowa, United States of America; National Institute of Infectious Diseases, Japan

## Abstract

**Background:**

Canine idiopathic inflammatory bowel disease (IBD) is believed to be caused by a complex interaction of genetic, immunologic, and microbial factors. While mucosa-associated bacteria have been implicated in the pathogenesis of canine IBD, detailed studies investigating the enteric microbiota using deep sequencing techniques are lacking. The objective of this study was to evaluate mucosa-adherent microbiota in the duodenum of dogs with spontaneous idiopathic IBD using 16 S rRNA gene pyrosequencing.

**Methodology/Principal Findings:**

Biopsy samples of small intestinal mucosa were collected endoscopically from healthy dogs (n = 6) and dogs with moderate IBD (n = 7) or severe IBD (n = 7) as assessed by a clinical disease activity index. Total RNA was extracted from biopsy specimens and 454-pyrosequencing of the 16 S rRNA gene was performed on aliquots of cDNA from each dog. Intestinal inflammation was associated with significant differences in the composition of the intestinal microbiota when compared to healthy dogs. PCoA plots based on the unweighted UniFrac distance metric indicated clustering of samples between healthy dogs and dogs with IBD (ANOSIM, p<0.001). Proportions of Fusobacteria (p = 0.010), Bacteroidaceae (p = 0.015), Prevotellaceae (p = 0.022), and Clostridiales (p = 0.019) were significantly more abundant in healthy dogs. In contrast, specific bacterial genera within Proteobacteria, including *Diaphorobacter* (p = 0.044) and *Acinetobacter* (p = 0.040), were either more abundant or more frequently identified in IBD dogs.

**Conclusions/Significance:**

In conclusion, dogs with spontaneous IBD exhibit alterations in microbial groups, which bear resemblance to dysbiosis reported in humans with chronic intestinal inflammation. These bacterial groups may serve as useful targets for monitoring intestinal inflammation.

## Introduction

Idiopathic inflammatory bowel disease (IBD) is a common cause of chronic gastrointestinal disease in dogs [1]. The most common clinical signs observed are chronic diarrhea and vomiting in combination with histopathologic evidence of intestinal inflammation [1]–[5]. While the exact etiology of canine IBD remains elusive, it is suspected that, similarly to human IBD (i.e., Crohn's disease and ulcerative colitis), the disease process is caused by complex interactions between environmental factors, the gastrointestinal microbiota, and an underlying genetic susceptibility of the host [6]–[8]. Most research evidence for a causal association between the enteric microbiota and host susceptibility as contributors to the pathogenesis of IBD is derived from rodent models and human IBD patients [9]. For example, in rodent models with an underlying genetic susceptibility (i.e., IL-10 gene deletion), intestinal inflammation develops only in the presence of bacteria but not in germ free animals. Furthermore, recent genome-wide association studies in humans affected by Crohn's Disease have revealed several susceptibility genes (e.g., NOD2/CARD15), which are implicated in defective bacterial killing as a consequence of impaired innate immunity [10]. Several recent molecular studies have revealed an altered microbial composition (i.e., dysbiosis) in the gastrointestinal tract of humans with IBD [11], [12]. The most commonly observed microbial changes are a decrease in Firmicutes, with a predominantly reduced diversity in *Clostridium* clusters XIVa and IV and a concurrent increase in the bacterial phylum Proteobacteria in affected patients [9], [11], [12].

Recent studies in dogs have identified genetic factors [13], [14] and compositional changes in the duodenum of dogs with idiopathic IBD. These dogs have significantly lower bacterial species richness and are significantly enriched in Proteobacteria compared to controls [6], [15]. However, these studies employed the construction of 16 S rRNA gene clone libraries, a labor-intensive method that typically allows only for analysis of a limited number of 16 S rRNA gene sequences, and it is therefore likely that changes in lower abundant microbial groups have remained uncharacterized. New molecular tools, such as 454-pyrosequencing, allow for deeper phylogenetic coverage of the intestinal microbiota and have demonstrated the presence of a highly complex gastrointestinal ecosystem in healthy dogs [16]–[18]. The aim of this study was, therefore, to characterize the duodenal microbiota of dogs with chronic idiopathic IBD using a 16 S rRNA gene pyrosequencing approach and to compare the duodenal microbiota to that in healthy dogs. Duodenal biopsies were selected as this part of the intestine is most commonly affected in canine inflammatory bowel disease.

## Materials and Methods

### Animals and Tissue Collection

Intestinal mucosal biopsies were collected via endoscopy from 14 dogs suspected of having either moderate (n = 7) or severe IBD (n = 7) based on a published clinical canine IBD activity index (CIBDAI) [3]. The CIBDAI is based on 6 criteria, each scored on a scale from 0–3: attitude/activity, appetite, vomiting, stool consistency, stool frequency, and weight loss. After summation, the total composite score is determined to be clinically insignificant (score 0–3), mild (score 4–5), moderate (score 6–8) or severe (score 9 or greater).

Endoscopic intestinal biopsies were also collected from control dogs (n = 6), which were free from gastrointestinal signs. This study had been approved by the Iowa State University (ISU) Institutional Animal Care and Use Committee. Informed consent to enroll clinical cases into the trial was obtained from each client.

Mucosal biopsy specimens were obtained from duodenal mucosa by endoscopy from each dog. A subset of mucosal specimens were placed in neutral buffered 10% formalin for H&E microscopy while another subset of biopsies (n = 4 per dog) were immediately placed in RNA stabilization solution (RNAlater, Qiagen) and kept at −80°C until isolation of RNA.

The control group was comprised of young adult, mixed-breed dogs of random-source origin that were free of gastrointestinal signs for at least six weeks prior to gastrointestinal sampling. These dogs were acquired directly from the Iowa State University (ISU) Laboratory Animal Resource facility as part of a pool of healthy control animals available for use in biomedical research. All dogs had previously been boarded for several months at local humane shelters since being abandoned by their owners. In this previous environment, they were housed individually, observed for clinical signs of illness as potential adoptees, and fed a commercially prepared adult dog maintenance diet (Teklad Laboratory Diet; 6.7 g of protein/100 kcal metabolizable energy (ME), 2.5 g of fat/100 kcal ME, and 12.7 g of carbohydrates/100 kcal ME) (Supplemental Table S1). After a several month timeline and due to housing shortages at the shelter, the dogs were then transferred to the ISU LAR rather than being euthanized at the shelter facility. Each dog was again examined for illness by LAR personnel, caged individually, and fed a commercial maintenance ration similar in composition to the previous shelter diet. They were boarded in the LAR area for approximately 3 months further and observed for gastrointestinal signs prior to study enrollment. None of the dogs were utilized as controls in other studies at ISU.

Medical records indicate that each control dog was dewormed with fenbendazole at recommended dosages and shown to be negative for *Giardia* spp. infection on repeated fecal examinations using zinc sulfate flotation techniques and direct fecal smears. No other medications, including antibiotics or anti-inflammatory drugs, were administered to any control dog prior to anesthesia for endoscopic examination. A thorough physical examination and laboratory evaluation consisting of a CBC, biochemistry profile, urinalysis, and GI panel at Texas A&M University (i.e., cTLI, cPLI, folate, and cobalamin concentrations) was performed on each dog to screen for underlying metabolic and systemic disorders not detected on physical examination.

Sample collection of endoscopic specimens for histopathologic analysis and RNA extraction was performed in an identical fashion and time frame to those diagnostic procedures performed on clinical IBD cases. A single experienced endoscopist (AEJ) performed gastroduodenoscopy on all animals. Twelve to fifteen small intestinal mucosal specimens were obtained from the mid to distal duodenum using standard serrated jaw pinch forceps passed through the 2.8 mm diameter accessory channel of the Olympus™ endoscope. The first 8–10 biopsy specimens collected on each dog were reserved for histopathology and the next 4 specimens were harvested for molecular analysis.

Clinical cases of canine chronic enteropathy were all referral cases into the gastroenterology service at ISU. A board-certified internist (AEJ, Diplomate ACVIM) served as the clinician-in-charge and oversaw the diagnostic evaluation of each dog with assistance from the veterinary students. Over the study period, 14 consecutive dogs having moderate-to-severe clinical disease activity were enrolled as previously described. This population of IBD dogs represented approximately 16% of the total canine chronic enteropathy caseload presented during the study period. Intestinal inflammation was scored on the basis of recently described histopathologic guidelines [19]. Mucosal biopsies from all dogs (both control and clinical cases) were reviewed in blinded fashion by a single experienced pathologist, board-certified by the American College of Veterinary Pathology (ACVP). All tissues were reviewed within 72 hours of collection and the results reported to the gastroenterologist (AEJ).

To reach a diagnosis of idiopathic IBD, all dogs underwent sequential therapeutic trials first with an elimination dietary trial followed by an antibiotic trial prior to endoscopic examination. To rule out adverse food reactions (e.g., food-responsive enteropathy) as the cause for gastrointestinal signs, each dog was fed a restricted antigen (i.e., novel intact protein) elimination diet for a minimal period of three weeks. Supplementary Table S1 summarizes the various diets the dogs received during this trial. The median (and range) amounts of macronutrient content across all diets were: 6.8 (4.5–7.5) g of protein/100 kcal ME; 4.1 (2.9–5.0) g of fat/100 kcal ME; 12.6 (7.9–15.0) g of carbohydrates/100 kcal ME. If the dog failed to respond to dietary intervention alone, an antibiotic (metronidazole, amoxicillin with clavulanic acid, or tylosin) was administered for 14 days. If dogs failed to respond to both dietary and antibiotic treatments, they were reevaluated to exclude other underlying disorders causing chronic gastroenteritis. All medications including antibiotics and corticosteroids were discontinued for at least 2 weeks prior to further diagnostic investigation. The study internist (AEJ) judged the clinical response to sequential treatments in each dog.

Diagnostic tests performed on IBD suspect dogs, which failed to respond to dietary or antibiotic interventions alone, included a CBC, serum chemistry profile, urinalysis, serum canine trypsin-like immunoreactivity (cTLI) concentration (Canine TLI radioimmunoassay, Siemens Medical Solutions Diagnostics), serum cobalamin and folate concentrations (Immulite 2000 Vitamin B12, Folic Acid, Siemens Medical Solutions Diagnostics), and serum C-reactive protein concentrations (CRP; Phase™Range Canine C-reactive Protein Assay, Tridelta Development Ltd). Further diagnostic testing included evaluation of feces for nematodes and protozoal parasites (i.e., wet mount and zinc sulfate flotation) and survey abdominal radiographs. Some dogs also had abdominal ultrasound performed for the evaluation of extra-alimentary tract abnormalities, such as renal and liver disease. Control dogs were evaluated in an identical fashion with the exception that diagnostic imaging and determination of CRP concentration were not performed.

### 454-pyrosequencing

Total RNA was extracted from the intestinal biopsies of each dog using the RNeasy total RNA midi kit (Qiagen®, Germany) according to the protocol outlined for RNA extraction from animal tissue. Extracted RNA was transcribed into cDNA using random monomers and the QuantiTect Reverse Transcription Kit with gDNA removal (Qiagen).

Bacterial tag-encoded FLX-titanium amplicon pyrosequencing (bTEFAP) based upon the V1–V3 region (*E. coli* position 27–519) of the 16 S rRNA gene was performed as described previously for canine intestinal samples at the Research and Testing Laboratory, Lubbock, TX, USA, with primers forward28F: GAGTTTGATCNTGGCTCAG and reverse519R: GTNTTACNGCGGCKGCTG [16], [18].

Raw sequence data were screened, trimmed, and filtered with default settings using the QIIME pipeline version 1.4.0 (http://qiime.sourceforge.net) [20]. Chimeras were detected and excluded using the software B2C2 (http://www.researchandtesting.com/B2C2.html) [21]. Operational taxonomic units (OTUs) were defined as sequences with at least 97% similarity using QIIME. For classification of sequences on a genus level the naïve Bayesian classifier within the Ribosomal Database Project (RDP, v10.28) was used. The confidence threshold in RDP was set to 80%.

### Statistical analysis

To account for unequal sequencing depth across samples subsequent analysis was performed on a randomly selected subset of 840 sequences per sample. This number was chosen to avoid exclusion of samples with lower number of sequence reads from further analysis. Alpha diversity (i.e., rarefaction) and beta diversity measures were calculated and plotted using QIIME. Differences in microbial communities between disease groups were investigated using the phylogeny-based unweighted Unifrac distance metric. This analysis measures the phylogenetic distance among bacterial communities in a phylogenetic tree, and thereby provides a measure of similarity among microbial communities present in different biological samples. To determine if any groups of samples (either based on clinical severity or severity of histological changes) contained significantly different bacterial communities, the analysis of similarities (ANOSIM) function in the statistical software package PRIMER 6 (PRIMER-E Ltd., Lutton, UK) was used on the unweighted UniFrac distance matrix [22]. To visualize clustering of samples by their relative abundance of bacterial families, a double dendrogram was generated using multivariate hierarchical clustering methods in NCSS 2007 (NCSS, Kaysville, Utah) [17].

Differences in the proportions of bacterial taxa (defined as percentage of total sequences) between healthy and IBD dogs were determined using non-parametric Mann-Whitney *U* tests (Prism5, GraphPad Software Inc.). Only taxa that were present in at least 50% of dogs (either healthy or diseased) were included in analysis. A Fisher's exact test was used to determine the proportions of dogs that harbored specific bacterial taxa. The resulting P-values were corrected for multiple comparisons on each phylogenetic level using the Benjamini & Hochberg's False Discovery Rate [23]. An adjusted P<0.05 was considered for statistical significance.

## Results

### Disease characteristics

The base-line clinicopathologic characteristics in IBD dogs (Table 1; Supplemental Table S1) were similar to previous reports [3]–[5]. In brief, dogs were predominantly middle-aged (median age 7.5, range 3–12 years) and exhibited chronic gastrointestinal signs of 3.6 months duration (range 1–36 months). None of the dogs had evidence of extra-alimentary tract inflammation based on results obtained from diagnostic testing. Seven dogs had moderate IBD (mean CIBDAI score = 6.5) and 7 dogs had severe IBD (mean CIBDAI score = 9.8). Four of 7 IBD dogs with moderate-to-severe clinical disease were diagnosed with protein-losing enteropathy, based on characteristic laboratory abnormalities. Endoscopic abnormalities of the small intestines (i.e., increased friability, granularity, and/or erosions) were observed in all IBD dogs. Histopathologic review of biopsy specimens showed that 7 dogs had mild, while 7 dogs had moderate-to-severe histologic lesions of IBD (Table 1).

**Table 1:** Baseline characteristics of study dogs.

	IBD dogs (n = 14)	Control dogs (n = 6)
**Sex**		
male, n (%)	6 (43)	3 (50)
female, n (%)	8 (57)	3 (50)
**Age**		
median (range), years	7.1 (3–12)	4 (3–6)
**Disease duration**		
median (range), months	3.6 (1–36)	0
**CIBDAI at endoscopy**		
median (range)	7 (6–12)	0
**Endoscopic lesions (%)**	100	0
**Histopathology**		
normal	0	6
mild IBD	7	0
moderate-to-severe IBD	7	0

CIBDAI = canine inflammatory bowel disease activity index.

IBD = inflammatory bowel disease.

The mean age of the healthy control (HC) dogs was 4 years (range 3–6 years). There were no abnormalities in the results of physical examination, CBC, serum biochemical analysis, urinalysis, multiple fecal examinations, dirofilarial antigen assay, endoscopic examination, or histopathologic findings of mucosal biopsies.

### Microbiota in the canine duodenum

**Figure 1:**
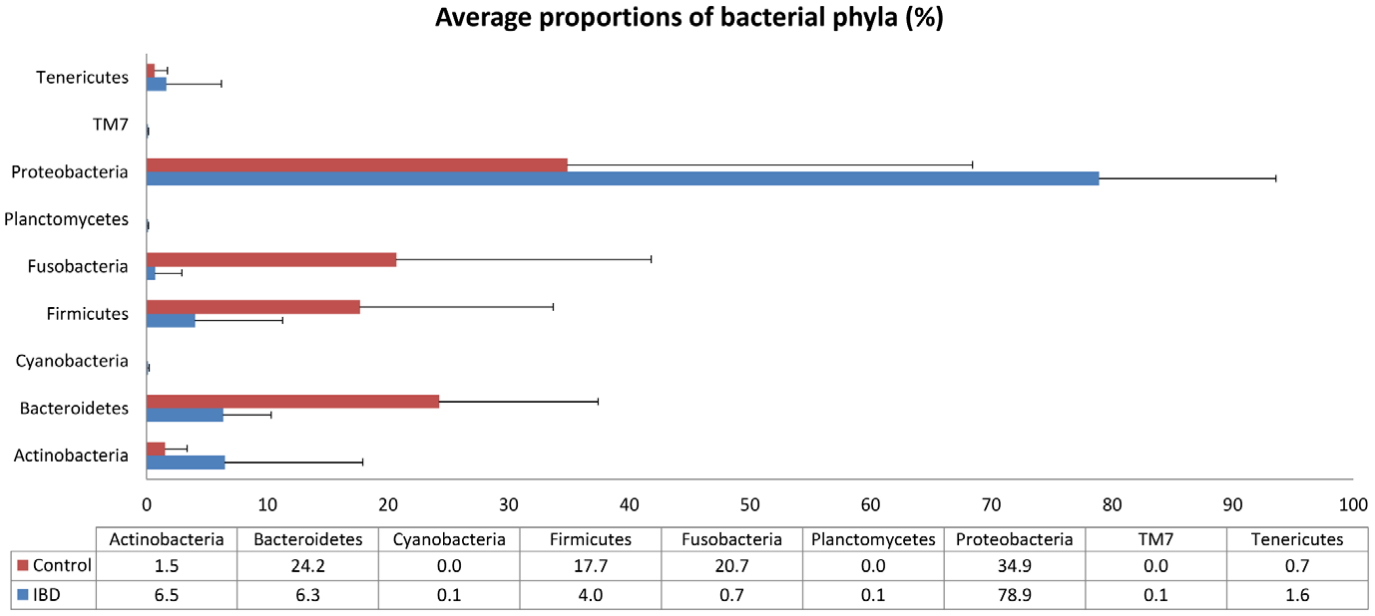
Average percentage of phyla identified in control dogs and dogs with IBD. Data represent the percentage of obtained total 16 S rRNA gene sequences. Error bars represent standard deviations.

**Figure 2:**
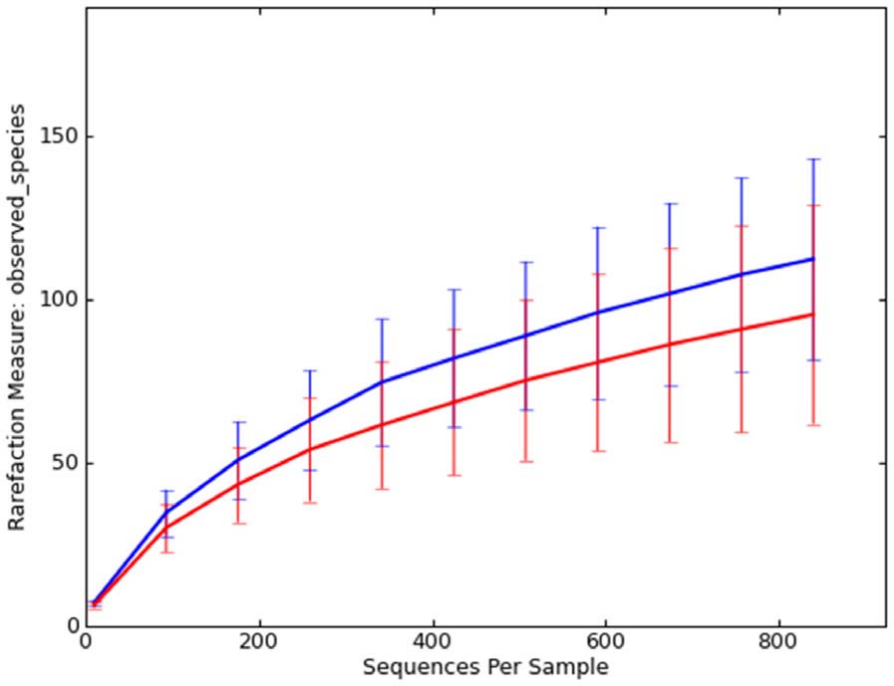
Rarefaction analysis of 16 S rRNA gene sequences obtained from canine duodenal mucosa samples. Lines represent the average of each group (blue = control dogs; red = dogs with idiopathic inflammatory bowel disease), while the error bars represent the standard deviations. The analysis was performed on a randomly selected subset of 840 sequences per sample.

The pyrosequencing pipeline yielded 73,998 quality 16 S rRNA gene sequences (mean 3,700 sequences per sample). A total of 9 bacterial phyla were identified across all control and IBD dogs (Fig. 1). Proteobacteria, Bacteroidetes, Firmicutes, Actinobacteria, and Fusobacteria represented more than 98% of all 16 S rRNA gene sequences. To avoid bias due to unequal sequencing depth, a subset of 840 randomly selected sequences were selected to calculate operational taxonomic units at 97% similarity (OTU_97_). No significant differences in median OTU_97_ were observed between disease groups: control group 112 (range 70–156); IBD group 84 (range 54–165); p = 0.4; Fig. 2)

### Microbial communities in controls and dogs with IBD

**Figure 3:**
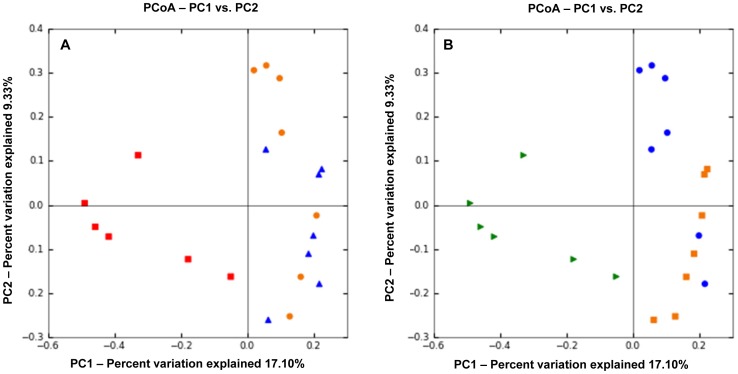
Principal Coordinates Analysis (PCoA) of unweighted UniFrac distances of 16 S rRNA genes. The clustering indicates differences in microbiota composition between controls and dogs with IBD. The analysis was performed on a randomly selected subset of 840 sequences per sample. Because the samples clustered along Principal Coordinates (PC) 1 and PC 2, only these graphs are shown. **A – Analysis according to clinical severity of disease based on the Canine Inflammatory Bowel Disease Activity Index (CIBDAI)**. Control dogs (red) separated from dogs with idiopathic inflammatory bowel disease (IBD; blue = moderate CIBDAI; orange = severe CIBDAI) indicating differences in microbiota ecology. No clustering is observed according to the severity of clinical disease. **B – Analysis according to severity of histopathology**. Control dogs with normal histology (green) separated from dogs with idiopathic inflammatory bowel disease (IBD; blue = mild changes on histology; orange = moderate-to-severe changes on histology). A trend was observed for separation between dogs with mild and dogs with moderate-to-severe histological changes (ANOSIM, p = 0.07).

**Figure 4:**
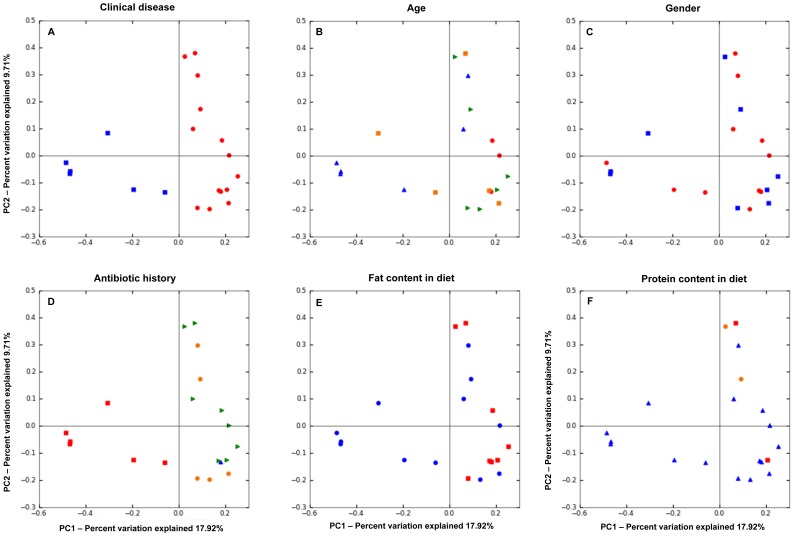
Principal Coordinates Analysis (PCoA) of unweighted UniFrac distances of 16 S rRNA. The analysis was performed on a randomly selected subset of 840 sequences per sample. The PCoA plots suggest that clustering was primarily based on intestinal disease rather than environmental factors. **A – Analysis according to clinical disease.** Control dogs (blue) cluster separately from dogs with IBD (red). **B – Age**. Blue symbols: dogs 3–4 years of age; orange: 5–6 years; green: 7–8 years; red: 12 years.**C – Gender.** Red = female dogs; Blue = male dogs. **D – Weeks between last dose of antibiotic administration and sample collection.** Green = 6 weeks; orange = 3 weeks; blue = 2 weeks; red = n/a (control dogs). **E – Fat content in diet.** Red = 2.5–4.0 grams of fat/100 kcal ME; blue = 4.1–5.0 grams of fat/100 kcal ME. **F – Protein content in diet.** Red = 4.0–5.9 grams of protein/100 kcal ME; blue 6.0–6.9 grams of protein/100 kcal ME; orange = 7.0–7.5 grams of protein/100 kcal ME.

Intestinal inflammation was associated with significant differences in the composition of the intestinal microbiota when compared to healthy dogs. PCoA plots (Fig. 3) based on the unweighted UniFrac distance metric indicated separation of samples between healthy dogs and dogs with IBD (ANOSIM, p<0.001), but no clustering was evident between dogs with moderate IBD and dogs with severe IBD as assessed by the CIBDAI (ANOSIM, p = 0.14). When clustering was assessed based on the severity of histological lesions, control dogs (normal histology) separated significantly from all IBD dogs (Fig. 3; ANOSIM, p<0.001). A trend was observed for separation between dogs with mild and dogs with moderate-to-severe histological changes (Fig. 3; ANOSIM, p = 0.07). Figure 4 indicated that the clustering was predominantly due to disease rather than to environmental factors such as gender, age, fat or protein content in diet, and antibiotic history.

Table 2 summarizes the relative proportions of the predominant bacterial taxa on various phylogenetic levels. Based on adjusted p-values, Fusobacteria were significantly decreased in IBD dogs (p = 0.01), while the decrease in Firmicutes (p = 0.095) and Bacteroidetes (p = 0.057), and the increase in Proteobacteria (p = 0.071) did not reach statistical significance. However, significant differences were identified within these phyla on lower phylogenetic levels (Table 2)

**Table 2:** Relative proportions of predominant bacterial taxa identified.

	Median % of sequences			No. of dogs harboring taxa		
	IBD * (min – max)	Control (min – max)	*P*-value^a^	IBD (n = 14)	Control (n = 6)	*P*-value^b^
**Phylum**						
Actinobacteria	1.20 (0.00–34.08)	0.79 (0.0–4.33)	0.433	13	6	1
Bacteroidetes	6.75 (1.51–26.01)	28.80 (1.64–37.04)	0.057	14	6	1
Firmicutes	4.1 (0.0–29.19)	15.29 (1.29–40.70)	0.095	12	6	1
Fusobacteria	0.00 (0.00–9.65)	14.56 (0.40–49.66)	0.010	5	6	0.07
Proteobacteria	72.54 (44.80–96.86)	31.58 (4.63–78.30)	0.071	14	6	1
**Class**						
Alphaproteobacteria	1.91 (0.04–22.62)	0.09 (0.00–56.45)	0.353	14	4	0.14
Bacilli	0.01 (0.00–23.07)	0.00 (0.00–3.14)	0.960	7	4	0.771
Bacteroidia	0.40 (0.00–25.14)	24.70 (1.60–36.41)	0.036	8	6	0.173
Betaproteobacteria	9.87 (1.08–31.68)	1.68 (0.20–36.97)	0.408	14	6	1
Clostridia	0.04 (0.00–12.50)	12.96 (0.78–40.43)	0.024	8	6	0.197
Deltaproteobacteria	0.42 (0.00–4.82)	0.00 (0.00–0.00)	0.068	9	0	0.056
Erysipelotrichi	0.00 (0.00–5.23)	0.10 (0.00–3.59)	0.042	1	4	0.034
Flavobacteria	1.90 (0.00–9.59)	0.32 (0.00–10.18)	0.578	12	4	0.72
Fusobacteria	0.00 (0.00–9.65)	14.56 (0.40–49.66)	0.040	5	6	0.042
Gammaproteobacteria	58.42 (10.91–94.36)	4.31 (0.30–50.29)	0.018	14	6	1
**Order**						
Actinomycetales	1.20 (0.00–34.08)	0.77 (0.00–4.32)	0.506	13	5	1
Aeromonadales	0.00 (0.00–0.00)	0.19 (0.00–3.57)	0.274	0	4	0.029
Bacteroidales	0.20 (0.00–7.17)	25.64 (2.59–38.86)	0.013	8	6	0.546
Burkholderiales	9.14 (1.08–31.68)	1.68 (0.011–36.97)	0.542	14	6	1
Caulobacterales	0.63 (0.00–22.62)	0.02 (0.00–2.49)	0.511	11	3	0.9
Clostridiales	0.00 (0.00–6.87)	12.81 (0.82–38.63)	0.019	6	6	0.265
Coriobacteriales	0.00 (0.00–0.00)	0.01 (0.00–0.25)	0.019	0	5	0.002
Enterobacteriales	8.56 (0.00–49.17)	0.23 (0.00–69.04)	0.464	13	5	0.5
Erysipelotrichales	0.00 (0.00–3.80)	0.07 (0.00–3.56)	0.295	0	4	0.029
Flavobacteriales	1.90 (0.00–9.59)	0.31 (0.00–10.18)	0.725	12	4	1
Fusobacteriales	0.00 (0.00–9.65)	14.56 (0.40–49.66)	0.010	5	6	0.33
Pseudomonadales	23.01 (0.16–91.41)	0.04 (0.00–1.48)	0.038	14	6	1
Rhizobiales	0.13 (0.00–7.75)	0.03 (0.00–9.78)	0.640	10	4	1
Sphingomonadales	0.00 (0.00–1.29)	0.04 (0.00–44.18)	0.447	5	4	0.89
Xanthomonadales	8.15 (2.32–39.12)	2.16 (0.01–7.39)	0.068	14	6	1
**Family**						
Alcaligenaceae	0.80 (0.00–10.69)	0.57 (0.08–1.73)	0.940	13	6	1
Bacteroidaceae	0.00 (0.00–3.93)	11.09 (0.00–36.27)	0.015	3	5	0.057
Bradyrhizobiaceae	0.00 (0.00–3.42)	0.03 (0.00–8.81)	0.365	5	4	0.454
Burkholderiaceae	0.27 (0.00–7.46)	0.00 (0.00–0.03)	0.084	10	1	0.12
Caulobacteraceae	0.63 (0.00–22.62)	0.01 (0.00–2.49)	0.330	11	3	0.482
Clostridiaceae	0.00 (0.00–10.81)	7.26 (0.54–23.38)	0.069	6	6	0.184
Comamonadaceae	5.90 (0.00–30.21)	0.14 (0.00–36.44)	0.349	13	4	0.338
Coriobacteriaceae	0.00 (0.00–0.00)	0.01 (0.00–0.25)	0.015	0	4	0.022
Enterobacteriaceae	8.56 (0.00–49.17)	0.23 (0.00–69.04)	0.144	13	5	1
Erysipelotrichaceae	0.00 (0.00–5.23)	0.10 (0.00–3.59)	0.044	1	4	0.077
Flavobacteriaceae	1.90 (0.00–9.59)	0.31 (0.00–10.18)	0.587	12	4	0.699
Fusobacteriaceae	0.00 (0.00–9.65)	14.56 (0.40–49.66)	0.055	5	6	0.154
Lachnospiraceae	0.00 (0.00–0.85)	2.85 (0.00–20.72)	0.044	3	5	0.079
Moraxellaceae	7.12 (0.00–45.96)	0.00 (0.00–0.00)	0.017	13	0	0.044
Prevotellaceae	0.00 (0.00–1.04)	3.60 (0.00–14.36)	0.022	3	5	0.066
Propionibacteriaceae	0.09 (0.00–34.08)	0.23 (0.00–1.11)	0.796	10	3	0.749
Pseudomonadaceae	20.89 (0.00–90.43)	1.01 (0.01–6.80)	0.051	13	6	1
Ruminococcaceae	0.00 (0.00–0.68)	0.46 (0.00–3.43)	0.044	2	4	0.102
Sphingomonadaceae	0.00 (0.00–1.29)	0.04 (0.00–44.18)	0.335	5	4	0.484
Succinivibrionaceae	0.00 (0.00–0.00)	0.19 (0.00–3.57)	0.045	0	4	0.068
Veillonellaceae	0.00 (0.00–0.00)	0.82 (0.00–2.84	0.004	0	4	0.033
Xanthomonadaceae	8.15 (2.32–39.12)	2.15 (0.01–7.39)	0.040	14	6	1
**Genus**						
Achromobacter	0.53 (0.00–10.69)	0.05 (0.00–0.23)	0.138	12	4	0.699
Acinetobacter	6.65 (0.00–48.43)	0.00 (0.00–0.01)	0.040	12	1	0.079
Allobaculum	0.00 (0.00–0.00)	0.01 (0.00–1.01)	0.183	0	3	0.132
Anaerobiospirillum	0.00 (0.00–0.00)	0.02 (0.00–3.57)	0.022	0	4	0.069
Bacteroides	0.00 (0.00–3.94)	11.25 (0.00–35.89)	0.088	3	5	0.198
Brevundimonas	0.23 (0.00–22.62)	0.01 (0.00–2.49)	0.339	10	3	0.674
Chryseobacterium	0.54 (0.00–6.04)	0.23 (0.00–2.70)	0.914	9	4	1
Citrobacter	0.57 (0.00–47.7)	0.00 (0.00–0.01)	0.060	10	0	0.079
Clostridium	0.00 (0.00–10.81)	7.26 (0.54–23.38)	0.026	6	6	0.113
Delftia	0.00 (0.00–10.12)	0.00 (0.00–3.05)	0.440	8	2	0.688
Diaphorobacter	1.85 (0.00–30.79)	0.00 (0.00–0.00)	0.088	11	0	0.044
Escherichia	0.49 (0.00–37.55)	0.05 (0.00–68.91)	0.238	12	5	0.733
Faecalibacterium	0.00 (0.00–0.04)	0.02 (0.00–3.43)	0.154	1	3	0.33
Fusobacterium	0.00 (0.00–9.69)	14.51 (0.39–49.51)	0.022	5	6	0.154
Megamonas	0.00 (0.00–0.00)	0.16 (0.00–2.72)	0.044	0	3	0.096
Prevotella	0.00 (0.00–0.78)	1.11 (0.00–10.20)	0.022	0	5	0.044
Propionibacterium	0.09 (0.00–34.08)	0.23 (0.00–1.11)	0.768	10	3	0.709
Pseudomonas	20.89 (0.00–90.43)	1.01 (0.01–6.80)	0.046	13	6	1
Ruminococcus	0.00 (0.00–0.10)	0.37 (0.00–6.07)	0.029	1	4	0.102
Sphingobacterium	0.00 (0.00–15.31)	0.00 (0.00–0.00)	0.237	6	0	0.211
Sphingopyxis	0.00 (0.00–0.04)	0.04 (0.00–42.98)	0.021	1	4	0.154
Sutterella	0.00 (0.00–0.00)	0.51 (0.00–1.62)	0.018	0	4	0.081

a Mann-Whitney Test;

b Fisher's exact test.

* *p*-values were adjusted for multiple comparisons based on the Benjamini and Hochberg False discovery rate.

Within Proteobacteria, the major changes were observed within the classes β- and γ-Proteobacteria. The genus *Diaphorobacter* (class β-Proteobacteria), a butyrate reducing bacterial group, was significantly more frequently identified in IBD dogs (11/14 dogs) than in controls (0/6 dogs; p = 0.044). Furthermore, Burkholderiaceae were also significantly increased in IBD. The class γ-Proteobacteria was increased in IBD, mostly due to increases in the order Pseudomonales and the families Moraxellaceae (genus Acinetobacter) and Xanthomonadaceae.

**Figure 5:**
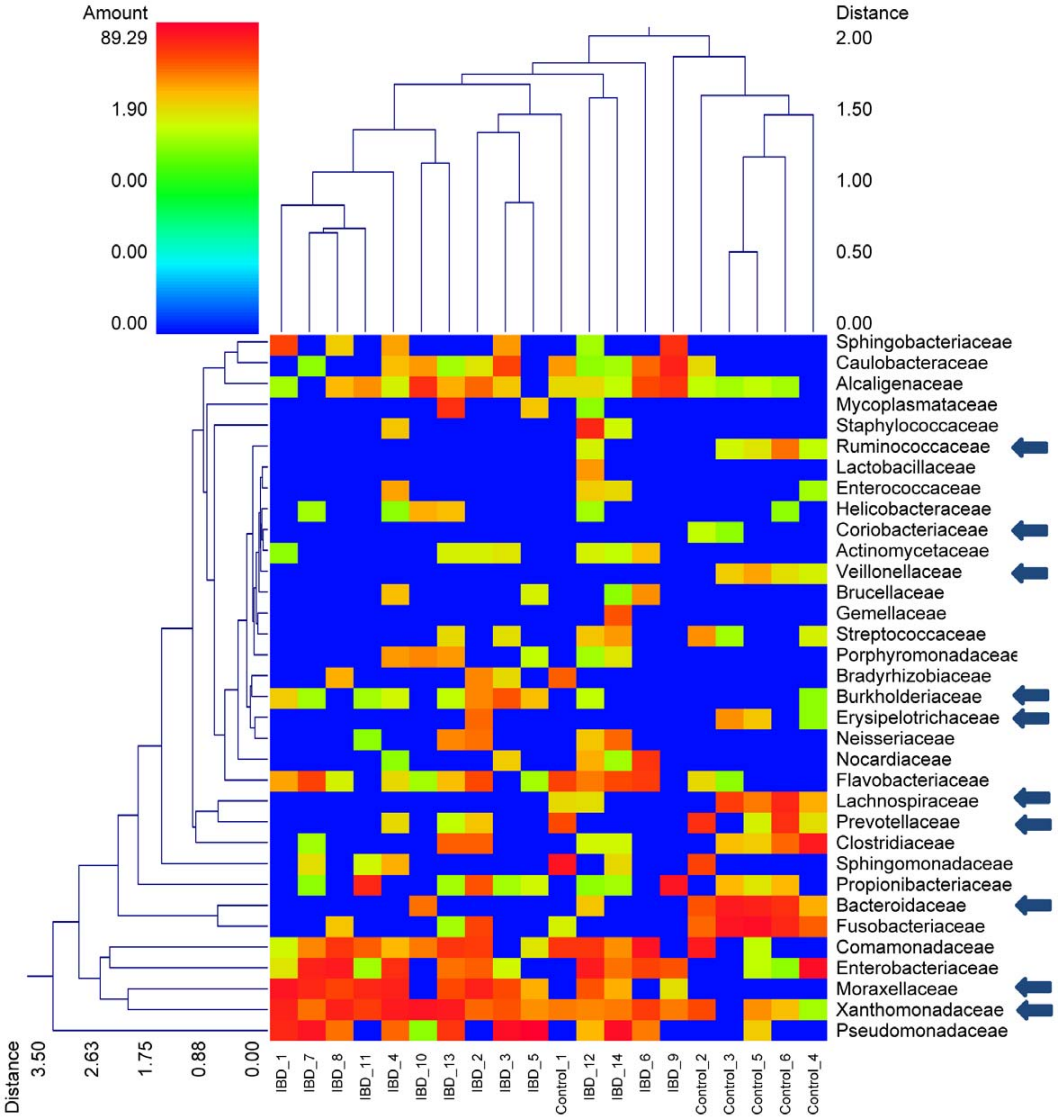
Dual hierarchal dendrogram based upon the predominant bacterial families. The clustering for bacterial families and for individual samples from the control dogs and dogs with IBD are based upon Furthest Neighbor metric with Euclidean distances. The arrows next to specific families denote significant differences in relative proportions observed between control and IBD dogs (based upon Mann-Whitney Test, see Table 2). The heatmap represents the relative percentage of each family within each sample with legend presented at the top left of the figure. Of note is that the dendrogram linkages of the bacterial families are not phylogenetic, but clustered based on relative family abundance among animals. Thus, those samples with more similarity (less distance) are more closely related in overall bacterial diversity. Similarly, those bacterial families which have similar percentages across all samples are more closely clustered.

Within the Firmicutes sequences belonging to Erysipelotrichaceae and to the Clostridiales were significantly decreased in IBD dogs (Table 2). For the latter group the decrease was mostly due to decreases in the families Ruminococcaceae (genus Ruminococcus), Veillonellaceae (genus Megamonas), and Lachnospiraceae (Table 2, Fig. 5).

Within the Bacteroidetes, the families Bacteroidaceae and Prevotellaceae (genus Prevotella) were also significantly decreased in dogs with IBD. No significant differences were identified for specific bacterial genera.

## Discussion

Idiopathic IBD in dogs is a commonly observed chronic enteropathy, which remains poorly understood with regards to disease pathogenesis. In humans, the current hypothesis for etiopathogenesis involves genetic (i.e., NOD2 polymorphisms) and environmental factors (e.g., enteric microbiota) which can cause dysregulated immune responses and drive chronic intestinal inflammation in susceptible individuals [9], [24]. There is emerging evidence that a similar etiopathogenesis also leads to chronic enteropathies in dogs. For example, granulomatous colitis in Boxer dogs is associated with the presence of adherent and invasive *Escherichia coli* (AIEC), and these AIEC isolates share phylogenetic and virulence similarities to AIEC isolates obtained from ileal tissue of humans with Crohn's disease (Simpson et al., 2006). Polymorphisms in TLR4 and TLR5 gene receptors have been associated with canine IBD [25]. Recent molecular studies have revealed a bacterial [6], [15] and fungal [26] dysbiosis in the duodenum of dogs with idiopathic IBD, as these dogs showed a significantly lower bacterial species richness (number of bacterial taxa detected) and were significantly enriched in Proteobacteria compared to controls. A recent study reported that German Shepherd dogs with chronic enteropathies have an altered expression of Toll-like receptors, which is associated with a unique and possibly breed-specific change in enteric microbiota composition [13].

In this study we used massive parallel pyrosequencing of the 16 S rRNA gene to evaluate potential differences in the mucosa-adherent microbiota between dogs with idiopathic IBD and control dogs. This automated high throughput technique allows a more thorough, in depth characterization of the intestinal ecosystem. Our results revealed that the canine duodenum harbors a complex intestinal assemblage, compromising at least 9 bacterial phyla. This is in contrast to previous molecular studies that have employed 16 S rRNA gene clone libraries and have revealed only 7 phyla in the canine duodenum [6], [27].

Our results demonstrate a dysbiosis in mucosa-adherent microbiota between healthy control dogs and dogs with IBD. Most notably, we observed an increase in sequences belonging to Proteobacteria, and a decrease in bacterial groups within Bacteroidetes, Fusobacteria, and Firmicutes. These results are in agreement with a previous study by our group, in which we have also observed increased proportions of sequences belonging to the various classes of Proteobacteria in duodenal mucosal biopsies from IBD dogs [6]. In this current study we have enrolled a new set of control and diseased dogs (i.e., none of the dogs analyzed here was part of the previous study), therefore confirming our previous results. Furthermore, we have employed a deep sequencing approach derived from RNA rather than DNA and we have identified additional groups that are altered in the duodenum of IBD dogs and possibly contribute to onset and perpetuation of chronic intestinal inflammation.

Our results are also in agreement with studies in humans with IBD, where similar patterns of dysbiosis are frequently observed. For example, humans with Crohn's disease consistently show a decrease in the bacterial phyla *Firmicutes* and *Bacteroidetes*, with a concurrent increase in *Proteobacteria* [12], [24]. Several studies evaluating the intestinal microbiome between controls and human IBD patients have further shown that within the Firmicutes, the *Clostridium* clusters XIVa and IV (i.e., *Lachnospiraceae*, *Ruminococcaceae*, *Faecalibacterium prausnitzii*, and *C. coccoides* subgroups) are consistently decreased in Crohn's disease patients [9], [12], [24]. These bacterial groups are believed to be the major producers of short chain fatty acids and therefore may play an important role in intestinal health. We have identified similar changes in our study, with decreases in Ruminococcaceae, Veillonellaceae, and Lachnospiraceae.

Other major phylogenetic groups that were associated with IBD were sequences of the β-and γ-Proteobacteria, which were significantly more ubiquitous in IBD dogs, compared with controls. Of interest was the finding that the genus *Diaphorobacter* (β-Proteobacteria) was more frequently identified in dogs with IBD. This genus contains bacterial species that are capable of reducing poly(3-hydroxybutyrate) and poly(3-hydroxybutyrate-co-3-hydroxyvalerate), however, the role of these compounds in gastrointestinal health and disease and their interactions with short-chain fatty metabolism is unknown at this point and requires further studies [28]. This bacterial group has not yet been associated with clinical disease in humans or animal species, and further investigations into the potential role of this bacterial group and its metabolic functions in the pathogenesis of canine IBD are warranted.

Despite many studies that have identified microbial dysbiosis in humans with gastrointestinal inflammation, the cause-effect relationship between microbial alterations and intestinal inflammation is not well understood. New hypotheses suggest that intestinal inflammation, due to for example transient *Campylobacter jejuni* or *Salmonella* infection, may trigger changes in mucosal architecture and in the innate immune reactivity, which in turn diminish the colonization resistance of the resident microbiota, resulting in an overgrowth of pathogens [29]. Furthermore, a depletion of commensal bacterial groups may lead to a reduced capability of the intestinal microbiome to down-regulate an aberrant intestinal immune response, leading to a perpetuation of intestinal inflammation. Counterbalancing this small intestinal dysbiosis with beneficial bacterial species (e.g. probiotics) may therefore be a reasonable objective in the treatment of canine inflammatory bowel disease in future studies.

A potential limitation of the present study may be the specific selection of control dogs. Our strict study inclusion criteria (disease free animals with normal diagnostic tests) resulted in a relatively small population of healthy control dogs. Due to ethical concerns it remains challenging to collect duodenal biopsy samples from a large cohort of healthy pet dogs. While laboratory-reared, age-matched Beagles were available for use as controls, we purposely chose to not utilize this population since their origin, medical histories, diet, and housing arrangements were not well characterized by the vendor. Also, we could not verify conclusively whether they had been used in other trials prior to arrival at ISU. Furthermore, it has been suggested that research-derived Beagles may harbor an altered intestinal microbiota and may have evidence of intestinal disease [30]. Therefore, we chose to enroll a smaller number of dogs of mixed-breed and of random source origin to serve as the control group.

It is important to acknowledge that environmental factors, such as age, dietary history and/or antibiotic history may have potentially biased our results. Figure 4 illustrates PCoA plots of unweighted Unifrac distances, examining the effects of these potentially confounding factors on the duodenal microbiota of control dogs and dogs with IBD.

There was a significant difference in age between the control dogs and dogs with IBD (median 4 years vs. 7.5 years, respectively; Table S1; p = 0.01). However, all dogs were adults and most dogs were considered middle-aged. Little is known about differences in microbiota composition at the different age stages of dogs. Based on previous culture-based results, neonatal puppies had a distinct microbial population in the first weeks to months of life that coincided with changes in diet and physiologic processes of the host [31]. One study compared research Beagle dogs of different ages (less than 12 months vs. more than 11 years of age) and found no significant differences in the cultivable bacterial groups in the stomach and small intestine [32]. A molecular fingerprinting study based on denaturing gradient gel electrophoresis (DGGE) showed no significant influence of age (dogs younger than 2.5 years vs. dogs older than 11 years) on canine fecal microbiota, although a trend was recorded [33]. Similarly, we did not observe any trend for clustering according to age in our study population (Fig. 4). With the advance in high-throughput sequencing methods it will be interesting to compare the various age groups in a larger population of dogs to examine the effect of age on the intestinal microbiome.

Diet is another potential confounding factor, as dogs where on various diets (Fig. 4). There was no statistical significant difference in protein and total carbohydrate content in the diets that were fed to the healthy dogs (median protein content: 6.7 g/100 kcal ME; median carbohydrate content: 12.7 g/100 kcal ME) and dogs with IBD (median protein content: 6.8 g/100 kcal ME; median carbohydrate content: 12.6 g/100 kcal ME 6.8 g/100 kcal ME). However, there was a significant difference in the fat content between groups (median fat content in the control group: 2.5 g/100 kcal ME; median fat content in IBD group: 4.1 g/100 kcal; p<0.001). Currently there is no consensus in veterinary literature as what levels of fat or protein would constitute a major difference in macronutrient content, and even less is known as what differences in macronutrient levels would be expected to cause a shift in the intestinal microbiome. While there is no published consensus, the fat content in the diets fed to control dogs and also dogs with IBD (2.5 g/100 kcal vs 4.1 g/100 kcal, respectively) may be considered typical for standard maintenance diets (http://vet.osu.edu/vmc/diet-manual). However, also other factors, including the digestibility of the diets, the substrate amount and the type entering the colon, and the type of carbohydrate present (fibers vs. starch) could alter the populations that reside in the gastrointestinal tract. How these factors affect the small intestinal microbiota as evaluated here remains unknown and warrants further studies. Based on our PCoA plots (Fig. 4) we did not observe clear trends based on fat content or protein content.

Studies have shown that administration of antimicrobials leads to alterations in the fecal microbiota of healthy humans [34]–[36]. These alterations may be noticeable already within 3–4 days of administration, and the quickness of return to the pre-treatment abundances of bacterial taxa is highly individualized. In some individuals, the microbiota started to return to pre-treatment state already within a few days after the end of administration [35], and in most individuals the microbiota resembled the pre-treatment state 4 weeks after the end of antibiotic administration [34]. However, some taxa failed to recover within a six month period [34]. While these studies have examined only healthy humans, similar results would be expected in canine fecal samples. Less is known about the effects of antimicrobials on small intestinal microbiota. In a previous study we have administered the macrolide antibiotic tylosin for 14 days to healthy Beagle dogs, and have evaluated the small intestinal microbiota before, at the end of administration, and 14 days after the end of administration [16]. Analysis using the Unifrac distance metric showed clustering of samples, which were collected during tylosin administration, but no clustering was observed 14 days after the end of antibiotic administration. The microbial diversity and also the microbial community resembled pre-treatment states in 3/5 dogs and 2/5 dogs, respectively. It is therefore, possible that antibiotic administration may have had an influence on the observed changes in microbial communities in our current study, as all dogs with IBD had a history of antibiotic administration. However, in eight of the 14 dogs with IBD the sample collection was performed more than 6 weeks after the last day of antibiotic administration, in 5 dogs samples were collected more than 3 weeks after antibiotic administration, and in the remaining dog the period between antibiotic administration and sample collection was approx. 2 weeks. While previous studies have shown that the intestinal microbiota is perturbed by antibiotic administration, it was generally observed that the microbiota returned to pre-treatment states within 4 weeks, although some groups were affected for longer periods of time [16], [34], [35]. We performed PCoA on the unweighted Unifrac distances to evaluate if clustering would be associated with the time period between the last day of antibiotic administration and sample collection (i.e., 6 weeks, 3 weeks, and 2 weeks; see Figure 4). Based on the above cited studies it would be reasonable to assume that a potential antibiotic effect would be less pronounced 6 weeks after cessation. Therefore, if the original observed separation between healthy dogs and dogs with IBD was due to an antibiotic effect rather than intestinal disease, it would be expected that at least those dogs with IBD that have not received antibiotics for more than 6 weeks would cluster closer to the healthy dogs. As Figure 4 illustrates, no clustering was obvious based on the duration of time between sample collection and antibiotic administration. Therefore, we speculate that the antibiotic effect is less likely to have caused the separation between healthy dogs and dogs with IBD as observed. However, it would be useful in future studies to compare a group of dogs with IBD to a control group that is matched according to different pre-treatments such as antibiotics and diet.

In future studies it will be also useful to adapt the standard protocols for DNA extraction based on the Earth microbiome Project (http://www.earthmicrobiome.org/emp-standard-protocols/), to allow for better comparisons of results across studies and laboratories. Paired samples, collected at the time of diagnosis and post-therapy, would have been informative to see if improvements in clinical signs are associated with changes in the duodenal microbiota. Furthermore, it will be useful in future studies to evaluate the role of parasites and viruses in the etiopathogenesis of chronic inflammatory diseases in dogs.

In conclusion, results of this study indicate that a complex mucosa-associated microbiota exists in the duodenum of dogs. Dogs with IBD have a dysbiosis, with the observed patterns of altered microbiota composition similar to those reported for humans with intestinal inflammation. As in humans with IBD, these altered bacterial groups represent potential targets for diagnosis and the development of better treatment modalities for canine IBD.

## Supporting Information

Table S1 **Characteristics of the dogs used in this study.**
(DOCX)
