# Supplementary material for: 16S rRNA Gene Pyrosequencing Reveals Bacterial Dysbiosis in the Duodenum of Dogs with Idiopathic Inflammatory Bowel Disease
Source: PLoS One. 2012 Jun 15;7(6):e39333. doi: 10.1371/journal.pone.0039333 (PMC3376104; doi:10.1371/journal.pone.0039333)
Supplement: Table S1 — Characteristics of the dogs used in this study. (DOCX) [file pone.0039333.s001.docx]

| **Supplemental Table 1**. Baseline characteristics of enrolled dogs | | | |  |  |  |  |  |
| --- | --- | --- | --- | --- | --- | --- | --- | --- |
| **Disease status** | **CIBDAI** | **Histotology** | **Age (yr)** | **Breed** | **Sex** | **Neuter status** | **Diet** | **Time since last antibiotic** |
| **IBD_1** | severe | moderate-severe | 8 | Australian Shepherd | M | Neutered | IVD duck and potato | > 6 weeks |
| **IBD_2** | moderate | mild | 3 | English Bulldog | F | Intact | IVD fish and potato | > 6 weeks |
| **IBD_3** | moderate | moderate-severe | 5 | Mixed breed | F | Neutered | IVD duck and potato | > 6 weeks |
| **IBD_4** | moderate | moderate-severe | 11 | Mixed breed | F | Neutered | IVD duck and potato | > 6 weeks |
| **IBD_5** | severe | mild | 8 | Jack Russel Terrier | M | Neutered | Hills i/d*# | > 3 weeks |
| **IBD_6** | severe | moderate-severe | 10 | Labrador Retriever | F | Neutered | IVD duck and potato | > 2 weeks |
| **IBD_7** | moderate | moderate-severe | 12 | Gordon Setter | F | Neutered | Iams low- residue | > 6 weeks |
| **IBD_8** | moderate | mild | 7 | Keeshond | M | Intact | Hills d/d | > 6 weeks |
| **IBD_9** | severe | moderate-severe | 8 | German Shorthair Pointer | F | Neutered | IVD fish and potato | > 3 weeks |
| **IBD_10** | moderate | moderate-severe | 7 | Keeshond | M | Intact | Royal Canin rabbit | > 3 weeks |
| **IBD_11** | moderate | mild | 6 | Shih Tzu | M | Neutered | Iams low-residue*# | > 3 weeks |
| **IBD_12** | severe | mild | 8 | Labrador Retriever | M | Neutered | Purina lamb and rice | > 6 weeks |
| **IBD_13** | severe | mild | 4 | Beagle | F | Neutered | Iams low-residue*# | > 3 weeks |
| **IBD_14** | severe | mild | 6 | German Shepherd | F | Neutered | Hills d/d | > 6 weeks |
| **Control_1** | healthy | normal | 6 | Mixed breed | F | Neutered | Teklad laboratory diet* | NA |
| **Control_2** | healthy | normal | 4 | Mixed breed | F | Neutered | Teklad laboratory diet* | NA |
| **Control_3** | healthy | normal | 3 | Mixed breed | F | Neutered | Teklad laboratory diet* | NA |
| **Control_4** | healthy | normal | 6 | Mixed breed | M | Neutered | Teklad laboratory diet* | NA |
| **Control_5** | healthy | normal | 3 | Mixed breed | M | Neutered | Teklad laboratory diet* | NA |
| **Control_6** | healthy | normal | 4 | Mixed breed | M | Neutered | Teklad laboratory diet* | NA |
| *Not an antigen-restricted diet; # previously failed one or more dietary trials with an antigen-restricted diet | | | | | | | | |
| IBD = inflammatory bowel disease; NA = not applicable; CIBDAI = canine inflammatory bowel disease activity index | | | | | | | |  |
| NA = not applicaple | |  |  |  |  |  |  |  |
